# Retrospective analysis of percutaneous intervention of the renal artery in transplanted kidneys in children and adolescents at a tertiary public hospital

**DOI:** 10.1371/journal.pone.0297975

**Published:** 2024-03-29

**Authors:** Ana Carolina Buso Faccinetto, Gustavo Rocha Feitosa Santos, Juliana Cristina Taguchi, Henry Campos Orellana, Attílio Galhardo, Gabriel Kanhouche, Manoela Linhares Machado Barteczko, Hélio Tedesco Júnior, Nathalie Jeanne Magioli Bravo-Valenzuela, Valdir Ambrósio Moises, José Osmar Medina Pestana, Célia Maria Camelo Silva, Adriano Henrique Pereira Barbosa

**Affiliations:** 1 Department of Medicine, Cardiology Division, Universidade Federal de São Paulo (UNIFESP), São Paulo, SP, Brazil; 2 Division of Nephrology, Hospital do Rim e Hipertensão, A Part of the Universidade Federal de São Paulo (UNIFESP), São Paulo, SP, Brazil; 3 Faculty of Medicine/ Federal University of Rio de Janeiro (UFRJ), Department of Pediatrics, Pediatric Cardiology Discipline, IPPMG, Rio de Janeiro, RJ, Brazil; Ataturk University Faculty of Medicine, TURKEY

## Abstract

**Background:**

This study evaluated the long-term effects of percutaneous intervention in children and adolescents with transplant renal artery stenosis (TRAS).

**Methods:**

Twenty patients had significant stenosis (>50%) and underwent percutaneous transluminal angioplasty (PTA/stenting) (TRAS group-intervention); 14 TNS (non-significant group -control) patients did not have significant stenosis (≤50%) and were treated clinically. The combined primary endpoints were death from all causes and late graft failure. The secondary endpoints were serum creatinine (SCr), systolic blood pressure (SBP), and diastolic blood pressure (DBP).

**Results:**

No statistically significant difference was found between TRAS—Intervention(N = 20) and TNS groups—Control (N = 14) for these clinical parameters: deaths, 1 (5.0%) vs. 0 (0.0%) (p = 1.000) and graft loss, 4 (20.0%) vs. 2 (14.3%) (p = 1.000). For the secondary endpoints, after 1 month and 1 year the values of SCr, SBP, and DBP were similar between the two groups but not statistically significant.

**Discussion:**

In the TRAS group (intervention), the stent implantation was beneficial for treating refractory hypertension and reducing blood pressure (BP) in children and adolescents. Despite the outcomes being similar in the two groups, it can be inferred that the patients in the TRAS group (intervention) would have had a worse outcome without the percutaneous intervention.

**Conclusion:**

TRAS treatment with stenting can be considered for children and adolescents. Because the sample in the present study comprised of only a specific population, further studies are needed for generalization.

**Trial registration:**

The trial was registered at clinictrials.gov with trial registration number NCT04225338.

## Introduction

Transplant renal artery stenosis (TRAS) can lead to hypertension and graft dysfunction [1]. Numerous studies specify percutaneous intervention as the treatment of choice for adult patients with TRAS [2]. TRAS is initially suspected on the basis of refractory hypertension and progressive graft loss in the absence of acute graft failure, calcineurin inhibitor toxicity, or urinary obstruction [3]. Depending on the definition and on the diagnostic techniques used, TRAS commonly manifests between the third month and the second year after transplantation [4].

The diagnosis of TRAS is based on clinical manifestations, complementary exams, or both. Post-transplant Doppler ultrasonography of the renal artery is the most used method because of its ease of access and low cost, and because it does not require the use of contrast or radioactive markers [5]. TRAS is suspected when the peak systolic velocity (PSV) on duplex ultrasonography is >200 cm/s with a resistive index of >0.50 [6].

The indication for pediatric renal transplantation and its treatment and complications are still under study [2]. TRAS accounts for 5%–15% of complications after pediatric kidney transplantation [2]. Factors associated with TRAS include the stenosis site, early diagnosis of graft dysfunction, cold ischemia time (CIT) (in the case of deceased donors), trauma of the donor vessels during surgery, surgical technique, different sites of renal artery stenosis, and time when transplantation was indicated [3]. These factors contribute to the occurrence of endothelial injury and inflammation due to ischemic lesions. The surgical manipulation is itself a contributing factor, as are atherosclerosis and infection by cytomegalovirus, which have mitogenic effects that trigger endothelial changes [3,7].

TRAS is the main vascular complication post transplantation and the long-term benefits and outcomes of percutaneous treatment in the pediatric population with TRAS are still being explored [3]. In this study, we evaluated the long-term clinical outcomes of children and adolescents with TRAS who underwent percutaneous intervention in a hospital specializing in kidney transplantation.

## Materials and methods

### Patient selection

The Hospital do Rim e Hipertensão is a part of the Universidade Federal de São Paulo (HRIM-UNIFESP), which is in São Paulo (the capital), Brazil, and specializes in kidney transplantation. In this single center has experience in performing kidney transplants in children and adolescents [8,9].

This is a sub-analysis of a retrospective case–control study approved by the Local Research Ethics Committee Universidade Federal de São Paulo [UNIFESP] and enrolled at clinictrials.gov with trial registration number NCT04225338. This study was conducted in accordance with Good Clinical Practice as per EU guidelines (EN 540), any local regulations, and the Helsinki Declaration. All medical ethics guidelines were followed throughout the research. The parents of all patients included in this study signed a consent form. In a time, interval of 7 years and 11 months (January 2007 to December 2014), at this single center (i.e., HRIM-UNIFESP) 36 children and adolescents with suspected with TRAS were studied were followed throughout the study. Two patients presented with complex stenosis and did not undergo angioplasty; they were, therefore, excluded from the study. Patients with suspected TRAS were first investigated for rejection and were excluded. These same patients who had refractory hypertension, deteriorating graft function, and an increase in PSV above 200 cm/s were referred for cineangiography of the transplanted renal artery.

All patients with suspected TRAS underwent angiography of the transplanted renal artery by the same team of interventional cardiologists. The patients were admitted on the previous day to the HRIM-UNIFESP transplantation unit and received aspirin and clopidogrel. Angiography was performed using the modified Seldinger technique after the puncture of the ipsilateral femoral artery. After image acquisition, the graft artery was cannulated selective, and image acquisition was again performed. Angiographic TRAS was defined as a reduction of > 50% in the lumen of the vessel, a pressure gradient ≥15 mmHg across the lesion, or both.

### Endovascular procedure

**Fig 1:**
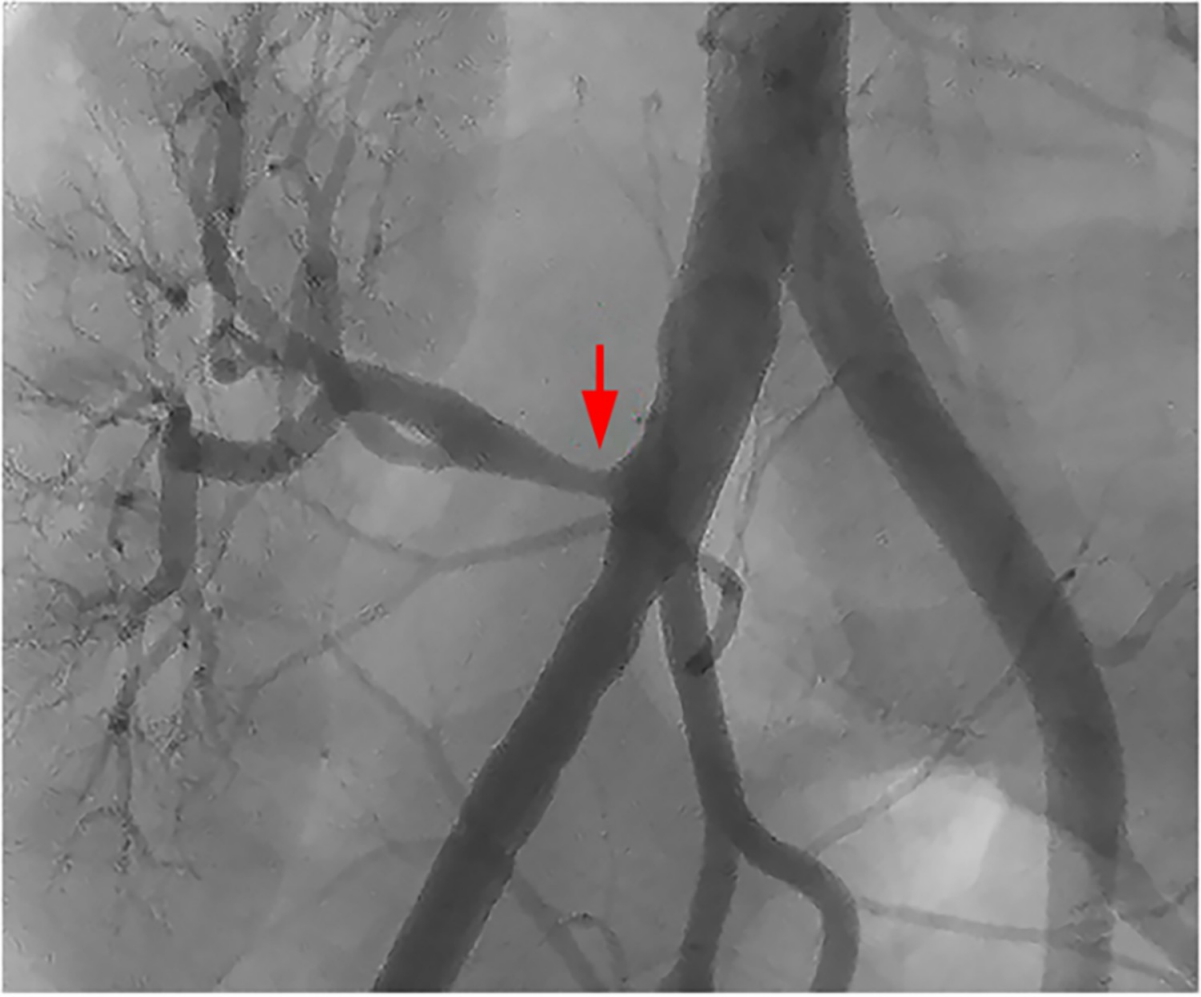
Angiography in TRAS group (intervention).

**Fig 2:**
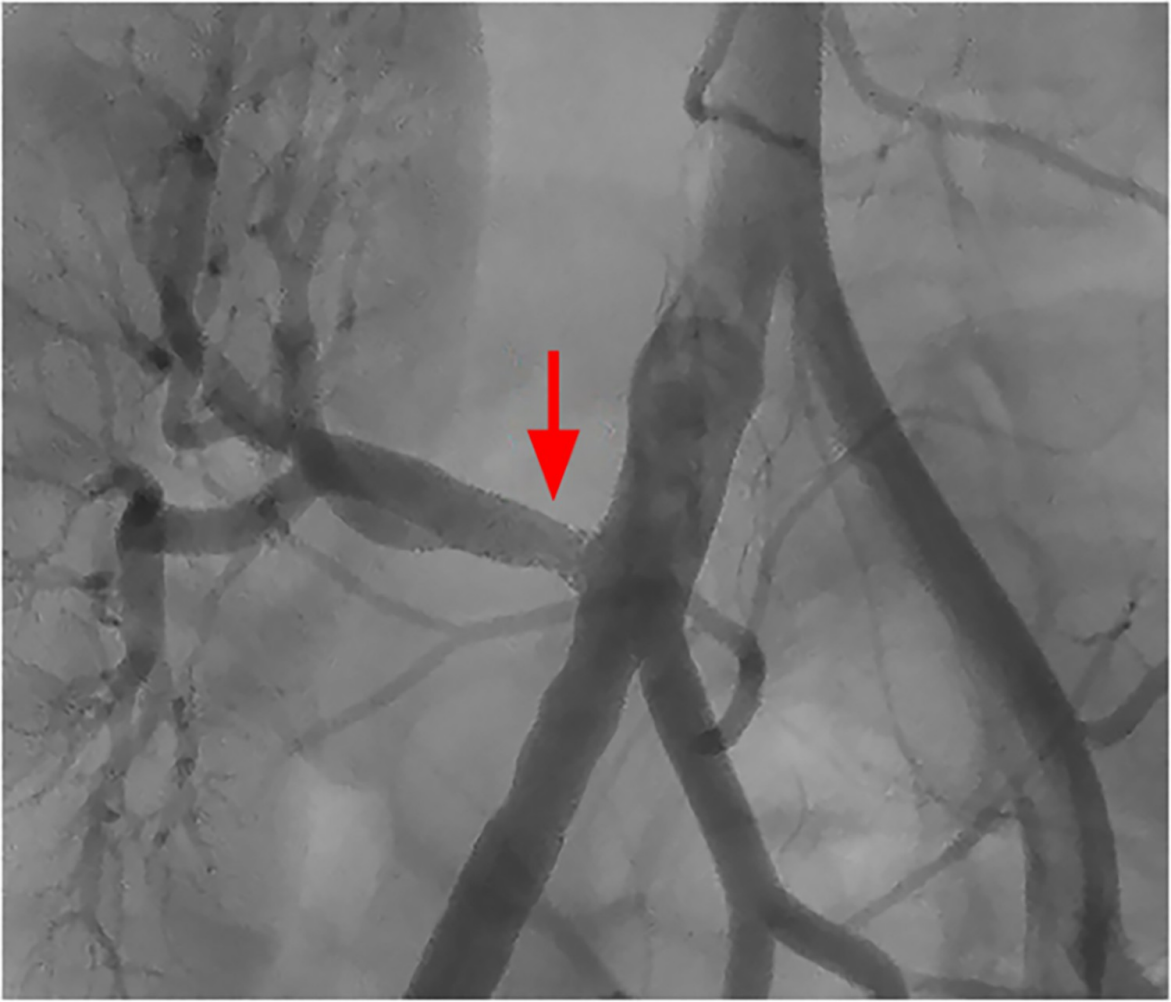
Intervention in TRAS group (intervention).

Patients with significant TRAS underwent an ad hoc intervention (Figs 1 and 2) in which unfractionated heparin (100 UI/kg IV) was administered and a 0.014" guide wire was passed through the stenosis.

**Fig 3:**
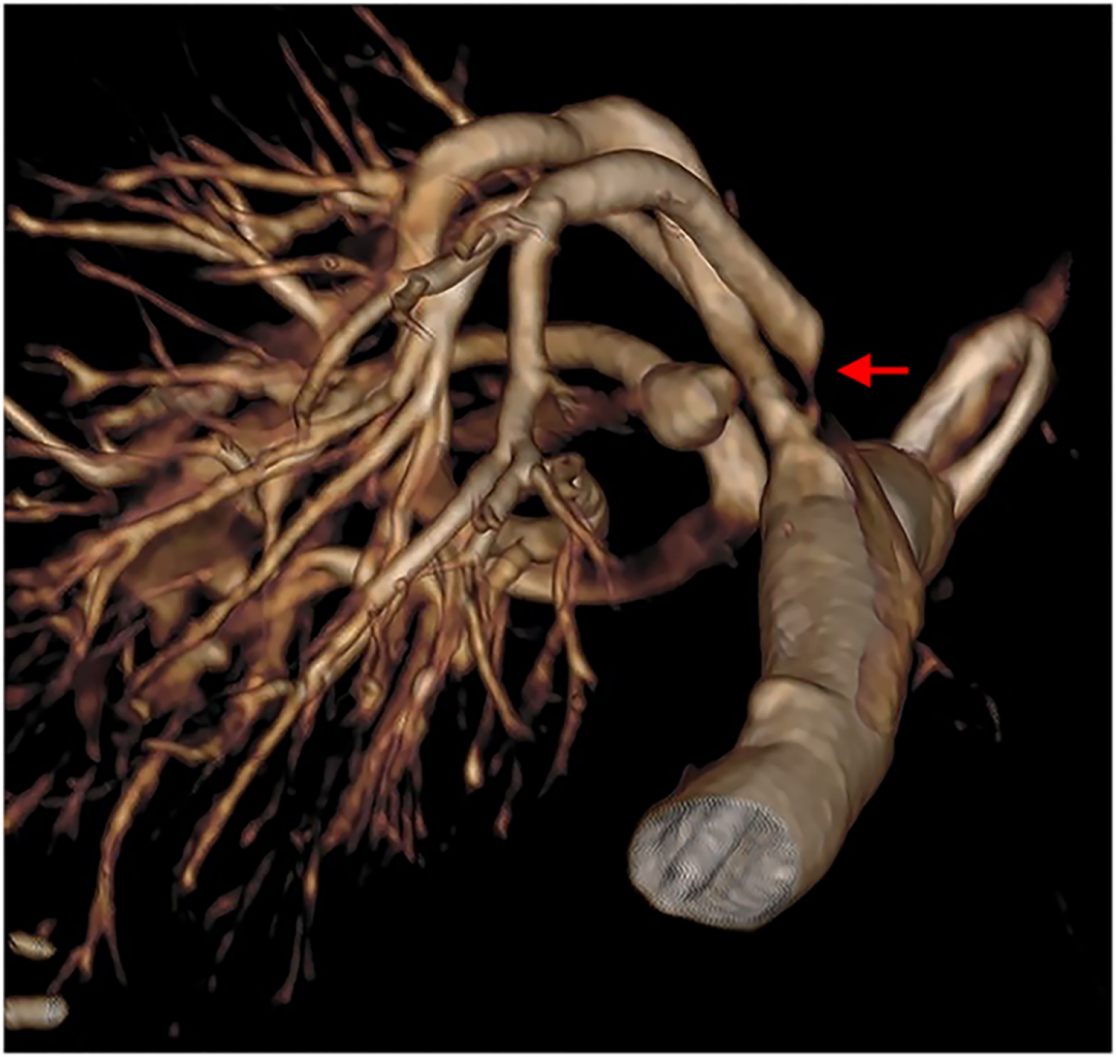
3D Angiography for analysis of stenosis and diagnostic complementation.

The dedicated renal stent was preferentially inserted directly without balloon dilation, except if the stenosis did not allow passing the stent or when the vessel’s diameter was less than 4 mm, in which case a coronary stent was used. The diameter of the balloon used ranged from 2.0 to 3.5 mm. In some cases, 3D angiography (Fig 3) was performed for diagnostic complementation.

The success of the procedure was considered complete if the residual stenosis was <20% without complication. The patients remained hospitalized for 24 h; on discharge, they were prescribed aspirin 100mg/day for 1 year and clopidogrel 75 mg/day for 1 month. The kidney transplant team conducted follow-ups.

### Data collection

The demographic, clinical, and follow-up data of patients who underwent angiography of the transplanted renal artery were collected directly from the medical records. The procedure data, success rates, and complications were recorded in the database of our catheterization laboratory.

The study data were collected from the medical record and managed using Research Electronic Data Capture (REDCap) electronic data capture tools (Vanderbilt University, Nashville, TN, USA) hosted at the Hospital São Paulo of the Universidade Federal de São Paulo (HSP-UNIFESP) [10,11]. All patients were de-identified. REDCap is a secure, web-based software platform designed to support data capture for research studies, providing 1) an intuitive interface for validating data capture; 2) audit trails for tracking data manipulation and export procedures; 3) automated export procedures for seamless data downloads to common statistical packages; and 4) procedures for data integration and interoperability with external sources. Patients with stenosis >50% who received the (Intervention -TRAS group) were compared to those who did not have stenosis or had stenosis ≤50% (TRAS nonsignificant [TNS] group-Control). Patients without long-term follow-up were excluded from the study.

### Endpoints of the study

The primary endpoints were defined as all-cause mortality and allograft survival. Based on the records of the kidney transplant team, graft loss was defined as the need for permanent dialysis. The secondary endpoints were defined as serum creatinine (SCr), glomerular filtration rate (GFR) by the Cockcroft-Gault formula, systolic blood pressure (SBP), and diastolic blood pressure (DBP) at 1 month and 1 year after angiography. The measurements were made in this specific population, according to the 7^th^ Brazilian Guideline on Arterial Hypertension [12]. Additionally, PSV and the number of antihypertensives were compared between the groups before angiography and at any moment during follow-up. The success rates of the procedure and the rate of restenosis were assessed. Complications were categorized according to the system of the Society of Interventional Radiology [13].

### Statistical analyses

The statistical analyses included a series of logistic regression models to predict death or kidney graft loss as the primary endpoints, using odds ratios and 95% confidence intervals to estimate the relative risk. The regression models were developed using a stepwise approach, with variables limited to three per step or per model. Moreover, the discriminatory ability of the models was evaluated using the Nagelkerke R^2^ test.

Data with a normal distribution were expressed as means ± SD (standard deviations), and not normally distributed data were expressed as medians (interquartile range [IQR]). Normal distribution and variations were assessed using histograms, the Kolmogorov–Smirnov test, normal probability plots, and residual scatter plots. To compare the baseline data, the chi-square test, the Mann–Whitney test, or two-tailed test. The statistical significance level was set at p < 0.05. The analyses were performed using the R package, version 4.0.0 (The R Foundation, Vienna, Austria).

## Results

Over a period of 7 years and 11 months, 367 pediatric kidney transplants were performed in the period, 36 children and adolescents (under ≤18 years of age) (Table 1) with suspected TRAS were admitted to the hemodynamics laboratory to undergo angiography for suspected TRAS. Two patients were excluded from the study: they had complex lesions and did not undergo angioplasty. Twenty patients with stenosis >50% underwent the intervention (TRAS group), and 14 patients had stenosis ≤50% (TNS group*- control*).

**Table 1:** Characteristics of the population submitted to angiography and pre-procedure.

*Total sample size (N = 34)*	N = 20 TRAS- Intervention	N = 14 TNS- Control	p	Test
Clinical characteristics				
Gender (%, male)	11 (55.0)	13 (92.9)	0.045	Chi square
Age (years) (median [IQR])	15.00 [7.90–17.00]	16.00 [13.00–18.00]	0.112	Mann–Whitney
Weight (kg, D0) (median [IQR])	45.00 [30.75–53.10]	50.85 [38.62–64.95]	0.236	Mann–Whitney
Height (cm, D0) (median [IQR])	160.00 [138.00–163.25]	162.00 [155.00–170.00]	0.354	Mann–Whitney
BMI (kg/m^2^, D0) (median [IQR])	18.53 [14.76–25.28]	18.25 [13.51–24.98]	0.828	Mann–Whitney
Time on dialysis (months) (mean SD)	14.85 (6.26)	9.93 (5.94)	<0.001	t-test
HLA class (%)			0.232	Chi square
1	8 (50.0)	7 (50.0)		
2	0 (0.0)	2 (14.3)		
Hypertension (%)	12 (60.0)	10 (71.4)	0.748	Chi square
Previous hypertensive nephropathy (%)	0 (0.0)	2 (14.3)	0.316	Chi square

Previous glomerulonephritis (%)	6 (30.0)	3 (21.4)	0.871	Chi square
Other previous causes—malformation of the genitourinary system (%)	7 (35.0)	5 (35.7)	1.000	Chi square
Undetermined cause of chronic renal disease (%)	7 (35.0)	4 (28.6)	0.983	Chi square
VAT median [IQR]) (min)	30.00 [23.00–38.00]	29.50 [23.25–32.25]	0.703	Mann–Whitney
Donor = Deceased (%)	20 (100.0)	11 (78.6)	0.120	Chi square
CIT (h) (median [IQR])	24.00 [20.25–27.09]	21.00 [18.15–25.00]	0.217	Mann–Whitney
Time since transplant (months) (median [IQR])	4.00 [3.00–8.00]	5.00 [4.00–13.00]	0.452	Mann–Whitney
Treatment for rejection (%)	8 (40.0)	6 (42.9)	1.000	Chi square
*Pre-procedure data*				
Pre-procedure creatinine (mg/dL) (median [IQR])	1.44 [1.22, 1.65]	1.33 [1.22, 1.54]	0.672	Mann–Whitney
Pre-angiography SBP mmHg (median [IQR])	140.00 [110.00, 150.00]	127.00 [120.00, 140.00]	0.628	Mann–Whitney
Pre-angiography DBP mmHg (median [IQR])	80.00 [80.00, 100.00]	76.00 [70.00, 85.00]	0.103	Mann–Whitney
Pre-procedure GFR (median [IQR])	55.00 [42.00, 60.00]	57.50 [49.50, 82.00]	0.255	Mann–Whitney
N of pre-procedure antihypertensive drugs (median [IQR])	2.00 [1.00, 3.00]	2.00 [1.00, 2.00]	0.543	Mann–Whitney
PSV * (median [IQR])	358.50 [340.25, 414.00]	440.00 [355.00, 481.00]	0.253	Mann–Whitney

IQR: Interquartile range; SD: Standard deviation; VAT: Vascular anastomosis time; CIT: Cold ischemia time; SBP: Systolic blood pressure; DBP: Diastolic blood pressure; GFR: Glomerular filtration rate; N: Number; PSV: Peak systolic velocity.

Considering the 20 children or adolescents (N = 34) with stenosis above 50% undergoing intervention or clinical treatment (control), the prevalence of Transplanted Kidney Artery Stenosis (TRAS) in these group was 58%.

In the article regarding the anthropometric, clinical and renal transplant characteristics of patients: There was no significant difference between the TRAS (intervention) and control groups regarding age, diagnosis of systemic arterial hypertension and body mass index (Table 1). The proportion of males was higher in the control group than in the TRAS group–intervention (Table 1). Mean systolic blood pressure and diastolic blood pressure in the TRAS group (intervention) were not significantly different in relation to the control group (p = 0.628 and 0.103, respectively) (Table 1). There was no significant difference between groups regarding the number of antihypertensive drugs used (p = 0.543) (Table 1).

There was no significant difference between the groups regarding the cause of chronic kidney disease and the proportion of the HLA class (Table 1).

The average time on dialysis in the TRAS group(intervention) was 14.85 (+/- 6.26) months, significantly longer than in the control group, which was 9.93 (+/- 5.94) months (p<0.001). There was no statistically significant difference between groups regarding the proportion of deceased donors, which occurred in 20 patients (100%) in the TRAS group (intervention) and in 11 (78.6%) in the control group (p = 0.120).

The organ preservation time was 30 (VAT/min) (23–38) in the TRAS group (intervention) and 29 (23.25–32.25) in the control group, with no significant difference between them (p = 0.703).

The organ cold ischemia time (CIT) was 24.00 (20.25–27.09) in the TRAS group (intervention) and 21.00 (18.15–25.00) in the control group, also without significant difference (p = 0.217) The median time from transplantation to angiography was 4 (3–8) months, with no significant difference in relation to the control group, which was 5 (4–13) months (p = 0.452).

About the diagnosis and treatment of stenosis of the artery of the transplanted kidney:

The diagnosis TRAS was made in all patients based on the performance of serial ultrasounds (Doppler) in the patients, associated with monitoring of blood pressure, GFR and creatinine indices.

The average peak systolic velocity of the transplanted kidney artery at Doppler ultrasound did not show a significant difference between the TRAS (intervention) and control groups (Table 1). At angiography, the median luminal reduction was 70% (70–80%) in the TRAS intervention group, significantly higher than in the control group, which was 30% (0–37.50%); p<0.001.

The most frequent location of the lesion was in the ostium of the artery of the transplanted kidney in 14 patients (70%) in the TRAS group (intervention) and in 5 patients (35.7%) in the control group (p = 0.103) (Figs 1 and 2).

Treatment with angioplasty was performed in the 20 patients in the TRAS intervention group (18 with stent implantation and 2 with balloon angioplasty only). Before stent implantation, there was pre-dilation in 19 patients. Mean stent diameters were 4.6 (± 1.09) mm and mean lengths were 12.67 (± 2.55) mm.

The type of stent used was the *Biotronic (Dynamic Renal Biotronik)* was used in 8 (44.4%) patients, *Coronary* stent in 6 (33.3%), and other models in 4 (22.3%). The median balloon pressure on the stent during implantation was 11 [10–12] atm.

Angioplasty with or without stent enlarged the vessel lumen quite satisfactorily in all patients, without residual stenosis (Fig 2). There was no residual stenosis and no complications occurred during and immediately after angioplasty with or without stent.

The following results were obtained for the primary endpoints (Table 2) in the TRAS intervention group (N = 20) and TNS- control group (N = 14) groups. Mortality was 1 (5.0%) vs. 0 (0.0%) (p = 1.000); the kidney graft loss was 4 (20.0%) vs. 2 (14.3%) (p = 1.000); and the composite endpoint was 5 (25.0%) vs. 2 (14.3%) (p = 0.742), with no statistical differences between the groups. There were no statistically significant differences in the secondary endpoints (Table 2).

**Table 2:** Primary and secondary endpoints.

		*Angioplasty of the Renal Artery*		
*Endpoints*	*Yes*	*No*	p	Test
	N = 20 TRAS Intervention	N = 14 TNS Control		
*Primary endpoints*				
Kidney graft loss (%)	4 (20.0)	2 (14.3)	1.000	χ2
Death (%)	1 (5.0)	0 (0.0)	1.000	χ2
Composite endpoint (%)	5 (25.0)	2 (14.3)	0.742	χ2
*Secondary endpoints*				
Creatinine after 1 month (mg/dL) (median [IQR])	1.23 [1.01–1.76]	1.40 [1.15–1.61]	0.785	Mann–Whitney
Creatinine after 1 year (mg/dL) (median [IQR])	1.25 [0.96–1.49]	1.42 [1.18–1.60]	0.234	Mann–Whitney
Delta creatinine after 1 month (mg/dL) (median [IQR])	-0.04 [−0.29–0.08]	−0.08 [−0.23–0.11]	0.846	Mann–Whitney
Delta creatinine after 1 year (mg/dL) (median [IQR])	−0.19 [−0.45–−0.10]	0.09 [-0.31–0.43]	0.234	Mann–Whitney
Drop >0.1 mg/dL in creatinine after 1 month (%)	14 (73.7)	7 (53.8)	0.435	χ2
SBP after 1 month (mmHg) (median [IQR])	127.50 [120.00–132.50]	120.00 [120.00–145.00]	0.827	Mann–Whitney
Delta SBP after 1 month (mmHg) (median [IQR])	−20.00 [−20.00–10.00]	5.00 [−8.25–15.50]	0.125	Mann–Whitney
Any drop in SBP after 1 month (%)	9 (52.9)	4 (33.3)	0.505	χ2
DBP after 1 month (mmHg) (median [IQR])	80.00 [70.00–90.00]	75.00 [70.00–82.50]	0.450	Mann–Whitney
Delta DBP after 1 month (mmHg) (median [IQR])	−10.00 [−20.00–0.00]	−3.00 [−11.00–2.50]	0.257	Mann–Whitney
Any drop in DBP after 1 month (%)	11 (64.7)	6 (50.0)	0.682	χ2
SBP after 1 year (mmHg) (median [IQR])	120.00 [120.00–135.00]	120.00 [110.00–130.00]	0.287	Mann–Whitney
Delta SBP after 1 year (mmHg) (median [IQR])	−21.50 [−30.00–8.75]	−7.00 [−18.00–0.00]	0.305	Mann–Whitney
Drop in SBP after 1 year (%)	7 (70.0)	5 (55.6)	0.861	χ2
DBP after 1 year (mmHg) (median [IQR])	80.00 [70.00–85.00]	70.00 [70.00–90.00]	0.695	Mann–Whitney
Delta DBP after 1 year (mmHg) (median [IQR])	−7.00 [−17.50–0.00]	0.00 [-17.00–14.00]	0.594	Mann–Whitney
Any drop in DBP within 1 year (%)	6 (60.0)	4 (44.4)	0.827	χ2
PSV after the procedure (median [IQR])	287.00 [260.50–371.50]	244.00 [198.50–409.00]	0.697	Mann–Whitney
Delta PSV (median [IQR])	−69.00 [−155.00–-13.00]	−116.00 [−223.50– −11.50]	0.697	Mann–Whitney
Any drop in PSV (%)	9 (81.8)	2 (66.7)	1.000	χ2
Glomerular filtration rate (GFR) within 1 month (median [IQR])	49.00 [32.50–64.00]	72.00 [52.50–77.50]	0.123	Mann–Whitney
GFR within 1 year (median [IQR])	58.00 [49.50–81.25]	70.50 [50.75–85.50]	0.373	Mann–Whitney
20% improvement in GFR within 1 month (%)	2 (16.7)	2 (16.7)	1.000	χ2
20% improvement in GFR within 1 year (%)	5 (41.7)	5 (38.5)	1.000	χ2
Number of de antihypertensive drugs after the procedure (median [IQR])	2.00 [1.00–2.50]	1.00 [1.00–2.50]	0.772	Mann–Whitney
Change in the absolute number of antihypertensives (median [IQR])	0.50 [0.00–1.00]	0.00 [0.00–0.75]	0.578	Mann–Whitney
Drop in the number of antihypertensives (%) χ2: Chi square;	2 (16.7)	2 (20.0)	1.000	χ2

Multivariate models (stepwise selection) and predictors were also created in the TRAS group (Table 3). Combined univariate models were created with the secondary endpoints (Table 3), (surrogates—Table 4) and the primary endpoints (death or kidney loss—Table 5).

**Table 3:** Univariate models, endpoints and multivariate model.

	*Odds Ratio*	*CI*	*p*	*R*^*2*^ *Nagelkerke*
**Surrogate endpoints at 1 year** Drop in creatinine > 0.1 mg/dL in the 1st year	0.95	0.72–1.26	0.736	0.004
Drop in DBP in the 1st year	0.96	0.72–1.23	0.831	0.002
Drop in SBP no 1st year	0.98	0.74–1.29	0.868	0.001
> 20% improvement in the GFR in the 1st year	0.73	0.56–0.94	0.023	0.158
**Combined surrogate endpoints** Combined surrogate endpoints in 1 month: integer points/maximum 5 points				
(drop in creatinine (> 0.1 mg/dL) or improved SBP or DBP or PSV or GFR):	0.85	0.73–0.98	0.036	0.135
Combined surrogate endpoints in 1 month ≧ 3 points vs. <3 points	0.76	0.57–1.01	0.064	0.107
Combined surrogate endpoints in 1 year: integer points/ maximum 5 points				
(drop in creatinine (> 0.1 mg / dL) or improved SBP or DBP or PSV or GFR):	0.90	0.80–1.02	0.102	0.085
Combined surrogate endpoints in 1 month ≧2 points vs. <2 points	0.75	0.54–1.04	0.096	0.088

PTA: Percutaneous transluminal angioplasty; HLA: Human leukocyte antigen; VAT: Vascular anastomosis time; CIT: Cold ischemia time; SBP: Systolic blood pressure; DBP: Diastolic blood pressure; PSV: Systolic peak velocity; GFR: Glomerular filtration evaluator.

**Table 4:** Surrogate endpoints.

Surrogate endpoints within 1 month				
Drop in creatinine >0.1mg/dL in the 1st month	OR 0.83	CI 0.64–1.09	*p* 0.186	*R*^*2*^ *Nagelkerke* 0.056
Drop in DBP in the 1st month	0.98	0.74–1.29	0.868	0.001
Drop in SBP in the 1st month	0.99	0.79–1.33	0.906	0.001
>20% improvement in the GFR in the 1st month	0.79	0.55–1.12	0.189	0.056
Drop in PSV	0.84	0.62–1.14	0.259	0.041
	*Odds Ratio*	*CI*	*p*	*R*^*2*^ *Nagelkerke*
**Surrogate endpoints at 1 year** Drop in creatinine > 0.1 mg/dL in the 1st year	0.95	0.72–1.26	0.736	0.004
Drop in DBP in the 1st year	0.96	0.72–1.23	0.831	0.002
Drop in SBP no 1st year	0.98	0.74–1.29	0.868	0.001
> 20% improvement in the GFR in the 1st year	0.73	0.56–0.94	0.023	0.158
**Combined surrogate endpoints** Combined surrogate endpoints in 1 month: integer points/maximum 5 points				
(drop in creatinine (> 0.1 mg/dL) or improved SBP or DBP or PSV or GFR):	0.85	0.73–0.98	0.036	0.135
Combined surrogate endpoints in 1 month ≧ 3 points vs. <3 points	0.76	0.57–1.01	0.064	0.107
Combined surrogate endpoints in 1 year: integer points/ maximum 5 points				
(drop in creatinine (> 0.1 mg / dL) or improved SBP or DBP or PSV or GFR):	0.90	0.80–1.02	0.102	0.085
Combined surrogate endpoints in 1 month ≧2 points vs. <2 points	0.75	0.54–1.04	0.096	0.088

DBP: Diastolic blood pressure; SBP: Systolic blood pressure; PSV: Peak systolic velocity; GFR: Glomerular filtration rater.

**Table 5:** Multivariate model (stepwise selection)—general model.

Combined endpoint: death or kidney graft loss				
Predictors	*Odds Ratio*	*CI*	*p*	*R*^*2*^ *Nagelkerke*
Hypertension (yes vs. no)	1.29	1.03–1.62	0.035	0.456
Site of stenosis: (Renal artery ostium [anastomosis])	1.32	1.06–1.63	0.016	
Site of stenosis: (Renal artery branches)	2.16	1.36–3.43	0.003	
**Univariate model (only predictors of the angioplasty group; N = 20 patients)**				
**Combined endpoint: death or kidney graft loss/predictors of the angioplasty group**				
Percent stenosis (integer percent points)	1.00	0.98–1.02	0.717	0.008
Pre-dilation (yes vs. no)	1.59	1.06–2.40	0.038	0.234
Implantation (yes vs. no)	0.73	0.45–1.18	0.217	0.091
Stent diameter (integer mm)	1.21	1.04–1.41	0.023	0.273
Stent length (integer mm)	0.98	0.92–1.06	0.669	0.011
Type of stent (coronary vs. Biotronik)	0.90	0.74–1.10	0.317	0.061
Stent pressure	1.02	0.97–1.07	0.548	0.022
Post-dilation (yes vs. no)	0.76	0.51–1.13	0.195	0.100
Flair	0.85	0.56–1.28	0.444	0.036
**Multivariate model (stepwise selection; predictors of the angioplasty group; N = 20 patients)**				
**Combined endpoint: death or kidney graft loss**				
Hypertension (yes vs. no)	1.31	0.99–1.74	0.081	0.657
Site of stenosis (Renal artery branches)	1.92	1.20–3.07	0.015	
Stent diameter (integer mm)	1.16	1.03–1.31	0.028	

## Discussion

TRAS is the main vascular complication post transplantation, with a wide range of incidences in the literature depending on the diagnostic method used. The cause of refractory hypertension was especially evident in the TRAS group (intervention) symptom of which it is evidenced as the first cause of graft dysfunction. In our study, all patients were treated with immunosuppressants and received prophylactic therapy for cytomegalovirus, with other causes being excluded.

Thus, as well as in the adult population, as evidenced in the study by *Rengel et al*. and other studies, TRAS was the main cause of hypertension in this group of children and adolescents [3,14].

In our study, there were no differences between the groups regarding the nutritional profile (since all patients were considered eutrophic on the z score), age, and HLA type, with the results being similar. None of the two groups presented contrast-induced nephropathy after cineangiography of the transplanted renal artery.

In the adult population, the main indication for renal transplant was complications from diabetes as demonstrated in the study by *Kanhouche G*. *et*.*al* [14]. Among children and adolescents (Table 1), the main indication for kidney transplantation was genitourinary system malformation, and the second indication was undetermined. Our findings were similar to other studies such as *Marchal et al*. in which genitourinary malformation was also an important cause for kidney transplantation on young population. [4,15].

In the TRAS group (intervention) (Table 1), time on dialysis, mean VAT, and CIT were longer than in the TNS group (control).

These high times are also evidenced in our population, as in the adult population, according to the study by *Halimi et al*., the increased period of ischemia can cause vascular, endothelial, and parenchymal damage leading to delayed graft function due to the production of oxygen-free radicals. Which may contribute to renal allograft dysfunction [16].

However, the mean time (months) since the transplant was shorter in the TRAS group, which demonstrates that patients with stenosis >50% present allograft dysfunction sooner. The donor was predominantly deceased in both groups, especially in the TRAS group (intervention). This type of donor is associated with more vascular complications, which may explain the occurrence of lesions requiring the implantation of a stent, these characteristics in our study were similar to those found in the adult population, as demonstrated by the study such as *Bull AS et al* [2]. No patient in the TRAS group (intervention) received a drug-eluting stent because this type of stent is not available in our service.

The characteristics of the patients who underwent angioplasty were treated patients presented stenosis >50% (N = 20), with most lesions being ≥70% (N = 16; 84.2%) (p < 0.001). The stenosis site was in the anastomosis ostium, and pre-dilatation was performed in half of the patients, using a stent with a mean diameter of 4.6 mm (1.09) [17,18]. In this study, univariate and multivariate models with combined endpoints of death or kidney loss were developed. Pre-dilatation and larger-diameter stents showed an association, and the stenosis sites (ostium and branches) were the predictors most related to mortality. A higher risk for the occurrence of TRAS in the child population, highlighted in the study by *Ghirardo et al*, would be the size of the vessels for performing the anastomosis, since in children the vessels are smaller, adjunctive risk factor represented by the diameter of the anastomosed vessels due to disproportional body weight between donor and recipient might indeed result in perianastomotic stenosis.

With regard to the primary endpoints of our study (Table 3), it can be inferred that restoring the lumen of the transplanted renal artery led to similar outcomes regarding allograft survival and patency in patients both with and without significant stenosis. The treatment of patients in the TRAS group (intervention) was successful with only one patient dying of a noncardiovascular cause.

In the study by *Ghirardo et al*, angioplasty was considered as a possible treatment for patients with TRAS, and the hypothesis of early treatment reducing cardiovascular events in children and adolescents was evaluated [3]. There are studies that evaluated cardiovascular and post-renal transplantation risk in children [7]. *Ghirardo et al* demonstrated an improvement in blood pressure indices and in the clearance of renal function after percutaneous treatment in children and adolescents [3]. All the secondary endpoints (surrogates) (Tables 4 and 5) evaluated in this study, namely, SCr, SBP, DBP, and GFR, 1 month and 1 year after treatment decreased or were similar relative to the values obtained using clinical treatment.

In the TRAS group (intervention), PSV and the number of antihypertensive drugs did not change relative to the clinical treatment. However, patients whose GFR improved by >20 mL/min/1.73 m^2^ within 1 year had a 27% lower risk (OR 0.73, CI 95%: 0.56–0.94) of kidney loss or death than those whose GFR did not improve.

An important component of the treatment for TRAS is clinical improvement in hypertension. Our study suggests benefit with stent angioplasty like other studies in children, such as *Repetto et al* [16].

The improvement of renal function of the graft, of pressure indices was also evidenced in the study of *Patel et al* used a 15% reduction in serum creatinine and a 15% reduction in diastolic blood pressure with no change in anti-hypertensive medication or a 10% reduction in diastolic BP with a reduction in anti-hypertensive medication to define clinical success in adult population [19].

In our study, we can say that the causes of stenosis are multifactorial and inflammatory factors are also involved in the process [20]. The pathophysiology of renal artery stenosis is complex. Numerous pathways such as the transforming growth factor-β (TGF-β) and fibroblast growth factor-23 (FGF-23) are part of the complex pathophysiology of atherosclerosis condition, kidney fibrosis and ischemic nephropathy. The vascular inflammation suppresses FGF signaling by reduction the expression of FGF receptor-1 (FGFR1), thereby increasing TGFβR1 expression and initiating endothelial to mesenchymal transition. Furthermore, some studies have linked high levels of transforming growth factor-β as a prognostic factor for poor response to cyclophosphamide in steroid-resistant nephrotic syndrome (SRNS). Despite the fact that steroid-resistant nephrotic syndrome is currently an important cause of progressive kidney disease, we had no cases in our sample [20,21]. The findings of our study and the pediatric and the adult population from the literature the benefits of percutaneous treatment were similar on both: TRAS group (intervention) showed similar evolutions with percutaneous treatment, such as an improvement in blood pressure levels, renal graft survival, decrease in the number of antihypertensive drugs [1–4,6,7,14,16–19,22,23].

Therefore, it can be speculated that patients with stenosis >50% may have had a worse outcome without treatment by percutaneous transluminal angiography. Thus, the stent implantation evidencedto be beneficial for treating refractory hypertension and reducing BP in children and adolescents; this result is consistent with that of other studies as well as demonstrated benefit in the treatment in the adult population [1,3–5,17]. These patients did not have a worse outcome than those who received clinical treatment. The stenoses in the latter group were ≤50%, because they were lesions considered non-serious by angiography and thus maintained in conservative treatment i.e., interventional treatment was not indicated, and the patients stayed on pharmacological treatment only.

The limitation of this study, as well as demonstrated in other studies such as *Ghirardo et al* was that it was a retrospective cohort. The secondary results were collected after 1 year. As the majority of the patients in the study are regularly assessed in this specialized center, and the long-term follow-up exposed more adverse events as well as benefits of clinical treatment compared with short-term follow-up [3,22,24]. Indeed, another limitation of this study is the small sample size due to the fact that it is a specific population (pediatric population).

## Conclusion

TRAS treatment with percutaneous intervention may be considered for the pediatric population with TRAS and can plays an important role in the contemporary management of medically refractory hypertension in children and adolescents. Further research long-term studies are required to monitor the progression of renal stenosis in this group of pediatric patients and to better evaluation of the results of treatment with drug-eluting stents.
